# The Challenge of Treating Complex Coronary Lesions: A Call for More Personalized Approaches in Angioplasty

**DOI:** 10.31661/gmj.v13i.3607

**Published:** 2024-10-19

**Authors:** Shirin Allord, Sina Mashayekhi

**Affiliations:** ^1^ Cardiovascular Research Center , Tabriz University of Medical Sciences, Tabriz, Iran

**Keywords:** Coronary Artery Disease, Angioplasty, Coronary Vessels, Stents, Precision Medicine

## Dear Editor,

Treating complex coronary lesions remains a significant challenge in modern interventional cardiology, especially in the context of angioplasty [1]. Despite advances in stent technology and procedural techniques, patients with bifurcation lesions, chronic total occlusions (CTO), and heavily calcified arteries continue to experience higher rates of restenosis, thrombosis, and adverse long-term cardiovascular outcomes [2][3] it is certainly one of the biggest challenges for interventional cardiologists. Methods. We present a retrospective analysis of patients from our center who underwent percutaneous coronary intervention (PCI). These suboptimal results reveal the need for a more personalized approach to the management of these patients [4].

Current treatment protocols often adopt a one-size-fits-all model that overlooks the critical anatomical variations and unique lesion characteristics of individual patients [4][5]. Advanced imaging technologies, such as intravascular ultrasound (IVUS) and optical coherence tomography (OCT), have shown significant potential in improving stent placement and optimizing patient outcomes [6][7]. A recent systematic review and network meta-analysis demonstrated that IVUS enhances long-term clinical outcomes compared to percutaneous coronary intervention (PCI), particularly by reducing the need for target lesion revascularization [8] IVUS, and OCT. The 2 coprimary outcomes were target lesion revascularization and myocardial infarction. The secondary outcomes included ischemia-driven target lesion revascularization, target vessel myocardial infarction, death, cardiac death, target vessel revascularization, stent thrombosis, and major adverse cardiac events. Frequentist random-effects network meta-analyses were conducted. The results were replicated by Bayesian random-effects models. Pairwise meta-analyses of the direct components, multiple sensitivity analyses, and pairwise meta-analyses IVI versus ICA were supplemented.RESULTS:The results from 24 randomized trials (15 489 patients: IVUS versus ICA, 46.4%, 7189 patients; OCT versus ICA, 32.1%, 4976 patients; OCT versus IVUS, 21.4%, 3324 patients). Also, a study by Ziad et al. reported that OCT guidance led to a greater minimum stent area compared to angiography guidance [9]. However, these tools are underutilized in routine practice [10]. Incorporating these imaging modalities into personalized treatment plans could enhance procedural accuracy and reduce the risk of complications [11].

Emerging research underscores the value of tailored treatment strategies. For instance, hybrid revascularization techniques and drug-eluting balloon therapy have demonstrated promise in treating complex lesions [12][13][14]. However, the inconsistent application of these methods, coupled with a lack of specific guidelines, has limited their broader adoption. It is imperative that interventional cardiology moves toward developing precise, patient-specific treatment strategies, leveraging these emerging technologies [13][15] further large-scale randomized controlled trials (RCTs).

Future research should prioritize the integration of advanced imaging techniques and personalized treatment into clinical practice [15] further large-scale randomized controlled trials (RCTs). Systematically incorporating these tools will allow for therapies that address the unique characteristics of each patient’s coronary lesions, ultimately improving long-term outcomes and reducing the risk of adverse events [12][15] alone or in combination with drug-eluting stents (DES).

We urge professional societies and regulatory bodies to update clinical guidelines and prioritize research that supports the transition toward precision medicine in angioplasty. This shift toward personalized approaches is essential not only for improving procedural outcomes but also for reducing complications and enhancing long-term survival rates. Ultimately, this transition will streamline resources, reduce healthcare costs, and significantly improve patient quality of life.

## Conflict of Interest

The authors have no competing interests to declare that are relevant to the content of this article.
